# How They Advertise in the Northwest

**Published:** 1899-08

**Authors:** A. V. Barnett

**Affiliations:** Annandale, Minn.


### HOW THEY ADVERTISE IN THE NORTHWEST.

### A SKILLED VETERINARY SURGEON.

A. V. Barnett, D.V.S., who has just returned from Kansas City, Mo., where he has been attending the Western Veterinary College since last fall, is now ready to promptly attend to all calls by telegraph, telephone, or mail, and they will be attended to. The doctor says the Western Veterinary College is one of the largest and best veterinary schools in the West. It has the largest clinic interducements in the world for the students. He is more than pleased with the knowledge that he has received from that school. He says he is well paid for his winter's work and he is now in good shape to perform any kind of surgical operations satisfactorily, and desires to say to the public that he will make a specialty of rigling, castenating, and all kinds of spraying, and in fact all kinds of surgery, and the treating of hog cholera, glanders, and tuberlin in cows, and especially in preventing horses cribbing and pulling on the bits and will stop a horse from hanging his tongue out of his mouth, and chronic lamenitus can be operated on where the horse is very lame. He says it can be cured. Horses with old chronic discharge wounds in the jaws or any part of the head, and cows that get downand can't get up, and in fact anything in disease of animals and in regard to horses teeth or teeth in four or five years old colts. There are thousands of horses and colts suffering from their teeth being out of masticating condition, can't chew their feed good and get poor and scrubby, don't geow and eat lots of grain and get poorer and poorer and can't perform the wants of their owners to any satisfaction, and other diseases are brought on, and they get wormy and lousy, and the owner loses the growth of his colts and the use of his horse which is suffering and is necessary to carry on his farm work. Loses pleasure driving and the pride of seeing his animals grow and looking sleek and glossy. But if the owner of such animals will call on me or bring the animals to me I will examine him, and put his mouth in good shape and it will put money in the owner's pockets and he will be proud of his animals. Call and give me a trial. It will pay every man owning a horse to call and have his horse's mouth examined before the spring work. If there is nothing the matter with the teeth no charge will be made to examine them. You can save grain enough in a month to pay for the work, and have the pleasure of seeing a nice, fat and slick horse. All orders will be promptly attended to. Call and give me a trial.

Yours truly, A. V. Barnett, D.V.S.,
Annandale, Minn.

—*The Wright County Times*, Monticello, Minn., March 30, 1899.

